# Ambient Air Pollution Exposures and Risk of Parkinson Disease

**DOI:** 10.1289/EHP135

**Published:** 2016-06-10

**Authors:** Rui Liu, Michael T. Young, Jiu-Chiuan Chen, Joel D. Kaufman, Honglei Chen

**Affiliations:** 1 Epidemiology Branch, National Institute of Environmental Health Sciences, National Institutes of Health, Department of Health and Human Services, Research Triangle Park, North Carolina, USA; 2 Department of Epidemiology, School of Public Health, University of Washington, Seattle, Washington, USA; 3 Department of Preventive Medicine, Keck School of Medicine, University of Southern California, Los Angeles, California, USA; 4 Department of Environmental and Occupational Health Sciences, and; 5 Department of Medicine, University of Washington, Seattle, Washington, USA

## Abstract

**Background:**

Few epidemiologic studies have evaluated the effects of air pollution on the risk of Parkinson disease (PD).

**Objective:**

We investigated the associations of long-term residential concentrations of ambient particulate matter (PM) < 10 μm in diameter (PM10) and < 2.5 μm in diameter (PM2.5) and nitrogen dioxide (NO2) in relation to PD risk.

**Methods:**

Our nested case–control analysis included 1,556 self-reported physician-diagnosed PD cases identified between 1995 and 2006 and 3,313 controls frequency-matched on age, sex, and race. We geocoded home addresses reported in 1995–1996 and estimated the average ambient concentrations of PM10, PM2.5, and NO2 using a national fine-scale geostatistical model incorporating roadway information and other geographic covariates. Air pollutant exposures were analyzed as both quintiles and continuous variables, adjusting for matching variables and potential confounders.

**Results:**

We observed no statistically significant overall association between PM or NO2 exposures and PD risk. However, in preplanned subgroup analyses, a higher risk of PD was associated with higher exposure to PM10 (ORQ5 vs. Q1 = 1.65; 95% CI: 1.11, 2.45; p-trend = 0.02) among women, and with higher exposure to PM2.5 (ORQ5 vs. Q1 = 1.29; 95% CI: 0.94, 1.76; p-trend = 0.04) among never smokers. In post hoc analyses among female never smokers, both PM2.5 (ORQ5 vs. Q1 = 1.79; 95% CI: 1.01, 3.17; p-trend = 0.05) and PM10 (ORQ5 vs. Q1 = 2.34; 95% CI: 1.29, 4.26; p-trend = 0.01) showed positive associations with PD risk. Analyses based on continuous exposure variables generally showed similar but nonsignificant associations.

**Conclusions:**

Overall, we found limited evidence for an association between exposures to ambient PM10, PM2.5, or NO2 and PD risk. The suggestive evidence that exposures to PM2.5 and PM10 may increase PD risk among female never smokers warrants further investigation.

**Citation:**

Liu R, Young MT, Chen JC, Kaufman JD, Chen H. 2016. Ambient air pollution exposures and risk of Parkinson disease. Environ Health Perspect 124:1759–1765; http://dx.doi.org/10.1289/EHP135

## Introduction

Air pollution is a complex and dynamic mixture consisting of particulate matter (PM), gases, organic components, and metals (Block et al. 2012). Numerous studies have consistently shown deleterious effects of air pollution on human health. Ambient PM, including PM_10_ (particles < 10 μm in aerodynamic diameter) and PM_2.5_ (particles < 2.5 μm in aerodynamic diameter), and nitrogen oxides (NO_x_) have been consistently associated with increased risk of pulmonary and cardiovascular diseases (Brook et al. 2004). Although results vary across studies, several epidemiologic studies report stronger associations among women than among men, particularly for older adults (Annesi-Maesano et al. 2003; Clougherty 2010).

Recent evidence suggests that air pollution may also adversely affect the integrity of the central nervous system (CNS) and may contribute to neurodegeneration through mechanisms such as chronic brain inflammation, oxidative stress, microglia activation, and white matter abnormalities (Block et al. 2012). For example, postmortem examination of brain tissues from residents in highly polluted urban areas showed elevated amyloid beta 42 (Aβ42) (Calderón-Garcidueñas et al. 2008, 2012), hyperphosphorylated tau protein (Calderón-Garcidueñas et al. 2012), and α-synuclein accumulations (Calderón-Garcidueñas et al. 2008); these alterations have been implicated in the pathogenesis of major neurodegenerative diseases such as Alzheimer disease and Parkinson disease (PD). Animal studies further demonstrated that exposure to concentrated ambient PM led to increased levels of α-synuclein in the midbrain (Levesque et al. 2011), loss of dopaminergic neurons in the substantia nigra (Veronesi et al. 2005), and elevation of proinflammatory factors in the brain (Levesque et al. 2011). Disruptions of the nasal and olfactory epithelial barriers were also reported in dogs exposed to urban air pollutants high in PM (Calderón-Garcidueñas et al. 2002, 2003). These changes closely resemble neuropathological alterations in the brains of PD patients.

Few epidemiologic studies have examined the potential roles of ambient air pollutants in PD, and findings are inconsistent (Finkelstein and Jerrett 2007; Kirrane et al. 2015; Palacios et al. 2014a, 2014b; Ritz et al. 2016; Willis et al. 2010). Therefore, we investigated the association between ambient PM_10_, PM_2.5_, and nitrogen dioxide (NO_2_) exposures and risk of PD in the Parkinson’s, Genes and Environment (PAGE) study.

## Methods

### Study Population

The PAGE study is a nested case–control study within the large prospective National Institutes of Health (NIH)-AARP (formerly known as the American Association of Retired Persons) Diet and Health Study. Detailed description of the NIH-AARP study has been described previously (Schatzkin et al. 2001). Briefly, the cohort was established in 1995–1996 by the National Cancer Institute to investigate the roles of diet and lifestyle in the development of cancers and other chronic diseases. The participants were 566,398 AARP members who were 50–71 years old and resided in one of six U.S. states (California, Florida, Louisiana, New Jersey, North Carolina, and Pennsylvania) or in one of two U.S. metropolitan areas (Atlanta, GA and Detroit, MI). At enrollment, participants completed a comprehensive baseline questionnaire on diet, demographic characteristics, health-related behaviors, and medical history.

### PD Case Identification and Control Selection

PD patients in the PAGE study were identified from the follow-up survey conducted during 2004–2006 among surviving participants in the original cohort. On the survey, participants reported whether they had ever received a physician diagnosis of PD and the year of their first diagnosis (in one of the following time periods: before 1985, 1985–1994, 1995–1999, or in or after 2000). A total of 2,432 participants reported a PD diagnosis. Controls (*n* = 3,548) were randomly selected from cohort participants who did not report a PD diagnosis on the follow-up questionnaire; controls were frequency-matched to cases by year of birth (in 5-year groups), sex, and race. Because residential information was collected at the baseline survey in 1995–1996, we excluded from our analyses 394 cases who reported a PD diagnosis before 1995. We also excluded 358 self-reported PD cases whose diagnosis was later denied by the patients themselves or by their treating physicians in the diagnostic confirmation procedure described below. We also excluded 124 self-reported cases with residential addresses that could not be geocoded (e.g., no valid ZIP code; Post Office boxes only; outside the United States). Of the controls, we excluded 221 with invalid addresses and 14 self-reported controls who were later confirmed by physicians to be cases. After these exclusions, we had a total of 1,556 PD cases with self-reported PD diagnosis in or after 1995 and 3,313 age-, sex-, and race-matched controls in the primary analyses. The PD case group included 700 physician-confirmed cases as described below and 856 self-reported cases that we were unable to reach for diagnostic confirmation. Cases without diagnostic confirmation were older at enrollment than physician-confirmed cases (64.3 ± 4.8 vs. 63.2 ± 4.9 years old) and were more likely to be ever smokers (58.3% vs. 52.5%), but they were less likely to be non-Hispanic whites (93.6% vs. 96.0%), to have college education or higher (44.6% vs. 56.1%), and to be physically active (≥ 5 times/week: 17.8% vs. 21.7%). However, they were not significantly different from confirmed cases with regard to sex, caffeine intake, residential region in the United States, and urban or rural setting.

Between 2007 and 2010, we contacted surviving PD patients to confirm the self-reported PD diagnosis. Detailed procedures have been published previously (Chen et al. 2010). Briefly, we asked the self-reported cases to confirm their reports and to permit us to contact their treating physicians. We then asked the treating physicians, mostly neurologists, to complete a diagnostic questionnaire and to send us a copy of the patient’s medical records pertaining to PD diagnosis. The medical records were subsequently reviewed by a movement disorder specialist from the research team (X. Huang, Department of Neurology, Pennsylvania State University-Milton S. Hershey Medical Center, Hershey, PA). A case was confirmed *a*) if the treating neurologist confirmed the diagnosis, or *b*) if the medical record included a final PD diagnosis or evidence of two or more cardinal signs of PD (with one being resting tremor or bradykinesia), a progressive course, responsiveness to dopaminergic treatments, and absence of features that suggested an alternative diagnosis. Of the 1,069 responses from physicians received, 940 (87.9%) PD diagnoses were confirmed.

### Exposure Assessment

We geocoded the primary addresses of study participants provided at the baseline survey (1995–1996) using ArcMap version 10 (ESRI, Redlands, CA) and used residential locations to estimate outdoor pollutant concentrations. Invalid addresses, such as those with no valid ZIP code, Post Office boxes, and addresses outside the continental United States were flagged and removed from the analysis. Daily PM_10_ and PM_2.5_ and hourly NO_2_ concentration data were obtained from the U.S. Environmental Protection Agency (EPA) (Air Quality System Data Mart, https://aqs.epa.gov/aqsweb/documents/data_mart_welcome.html; see also Novotny et al. 2011; Sampson et al. 2013) ambient air pollution-monitoring stations in the contiguous United States, described in detail elsewhere (Novotny et al. 2011; Sampson et al. 2013). Average ambient PM_10_ and PM_2.5_ concentrations for the years 1990 and 2000, respectively, were estimated at the residential locations using a regionalized national universal kriging model (Sampson et al. 2013). Ambient NO_2_ concentrations for the year 2006 were estimated using a land-use regression model that combined data from fixed-site ambient monitoring stations, satellite-derived ground-level measurements, and land-use measurements (Novotny et al. 2011). The cross-validated *R* ^2^ value for the PM_2.5_ model for the year 2000 was 0.88, as previously reported (Sampson et al. 2013). In addition to residential information, the baseline survey also collected information on demographics and lifestyle factors including age, sex, race, smoking status, caffeine intake, and physical activity.

### Statistical Analysis

We estimated multivariate odds ratios (ORs) and 95% confidence intervals (CIs) from unconditional logistic regression performed separately for PM_2.5_, PM_10_, and NO_2_ concentrations. Because little is known about the association of these air pollutants with PD, we defined exposures both in quintiles and as continuous variables. Covariates included baseline age (in 5-year groups), sex, race (whites vs. nonwhites), smoking status (never smoker vs. ever smoker), caffeine intake (quintiles), region of the United States (northeast, midwest, west, south), and physical activity (never or rarely, times/week: < 1, 1–2, 3–4, and ≥ 5). Because epidemiological studies have consistently observed a lower risk of PD among smokers (Chen et al. 2010) and have suggested differences in both PD incidence (Haaxma et al. 2007) and risk factors (Weisskopf et al. 2007; O’Reilly et al. 2010; Chen et al. 2002) by sex, we performed additional analyses stratified by sex and baseline smoking status, and we tested for potential interactions by including a multiplicative interaction term in the regression model. We examined the statistical significance for a linear trend by including a continuous variable defined by the median value of each pollutant exposure quintile in the regression model. Because these analyses indicated associations among women and never smokers, we further conducted post hoc analyses and examined the association between each pollutant exposure and PD risk exclusively among female nonsmokers. Among female nonsmokers, we conducted analyses further stratified by moving status, defined as whether the participant maintained the same latitude and longitude coordinates between baseline and follow-up surveys. The primary analyses were conducted with all eligible cases, but we also performed sensitivity analyses limited to physician-confirmed cases. Finally, we performed additional sensitivity analyses stratified by census regions (U.S. Census Bureau; http://www2.census.gov/geo/pdfs/maps-data/maps/reference/us_regdiv.pdf) of the United States. We recategorized the pollutant exposures into tertiles to preserve sample sizes in exposure categories. We did not perform this analysis for the Midwest region owing to the very small sample size. All statistical analyses were perfomed using SAS v.9.1 (SAS Institute Inc., Cary, NC). Significance tests were two-tailed, with α = 0.05.

### Standard Protocol Approvals, Registrations, and Patient Consents

Participants consented to the study by returning survey questionnaires. The study protocol was approved by the Institutional Review Board of the National Institute of Environmental Health Sciences and the Special Studies Institutional Review Board of the National Cancer Institute.

## Results

Baseline characteristics of the study population according to PD diagnosis are presented in Table 1. Compared with those without PD, PD cases were more likely to be men and to have college-level education or above; they were also more likely to be never smokers and to have lower caffeine intake. The majority of the PAGE participants lived in urban counties, and the percentage of urban versus rural dwellers was comparable for PD cases and the selected controls. PM_2.5_ was moderately correlated with PM_10_ and NO_2_ (*r* = 0.57 and 0.62, respectively), and NO_2_ was also moderately correlated with both PM_2.5_ and PM_10_ (*r* = 0.58 for both).

**Table 1:** Baseline characteristics of study participants in the National Institutes of Health (NIH)-AARP Diet and Health Study according to Parkinson disease diagnosis after 1995.*^a^*

Characteristic	No PD	PD
*n*	3,313	1,556
Mean age, years (SD)	63.5 (4.8)	63.8 (4.9)
Men, %	73.5	74.2
Race, %
Non-Hispanic white	94.4	94.7
Other	4.7	4.6
Missing	0.9	0.7
Education, %
< 12 years	20.7	20.5
High school	9.3	6.8
Some college	21.5	20.5
College and above	46.3	49.8
Missing	2.2	2.3
Physical activity, %
Never or rarely	15.3	15.3
1–3 times/month	12.2	12.5
1–2 times/week	21.3	19.3
3–4 times/week	28.9	32.5
≥ 5 times/week	21.3	19.5
Missing	0.9	0.9
Caffeine intake (mg/day), median (IQR)	235.8 (528.7)	194.0 (522.8)
Smoking status, %
Never	35.2	43.2
Past	55.7	50.6
Current	8.0	5.1
Missing	1.1	1.1
Region of United States, %
Northeast region	27.7	28.3
Midwest region	5.3	5.3
West region	31.5	33.1
South region	35.5	33.2
Residential setting, %
Urban	92.0	92.9
Rural	8.0	7.1
Abbreviations: IQR, interquartile range (25–75%); PD, Parkinson disease; SD, standard deviation. ^***a***^Percentage may not sum to 100 because of missing values.		

Overall, we found no statistically significant associations between exposures to ambient PM_10_, PM_2.5_, or NO_2_ and PD risk (Table 2). In *a priori* analyses stratified by sex (Table 3), high exposure to PM_10_ was associated with an increased risk of PD among women (OR_Q5 vs. Q1_ = 1.65; 95% CI: 1.11, 2.45; *p*-trend = 0.02) but not among men (OR = 0.92; 95% CI: 0.73, 1.14; *p*-trend = 0.95). In *a priori* analyses stratified by smoking (Table 3), exposure to the highest quintile of PM_2.5_ concentration was associated with an increased risk of PD among never smokers (OR_Q5 vs. Q1_ = 1.29; 95% CI: 0.94, 1.76; *p*-trend = 0.04). However, the *p* for interaction was not statistically significant in either analysis. Additional adjustment for U.S. region modestly attenuated the associations without materially changing the results (data not shown). Further, in the analyses stratified by U.S. regions, a higher risk of PD was observed in the South for exposures to the highest tertiles of PM_2.5_ and PM_10_ among females and for exposures to the highest tertile of PM_2.5_ among never smokers (see Table S1). In post hoc analyses among female never smokers (Table 4), a higher PD risk was associated with increased exposures in the top two quintiles of PM_2.5_ (OR_Q4 vs. Q1_ = 2.38; 95% CI: 1.32, 4.31 and OR_Q5 vs. Q1_ = 1.79; 95% CI: 1.01, 3.17; *p*-trend = 0.05) and in the top quintile of PM_10_ (OR_Q5 vs. Q1_ = 2.34; 95% CI: 1.29, 4.26; *p*-trend = 0.01). Additional adjustment for region did not materially change the results. Further sensitivity analysis stratified by U.S. region showed a significantly higher risk of PD with high PM exposures among individuals living in the South (see Table S2). For all analyses, weak positive associations were also found when PM_2.5_ and PM_10_ exposures were defined as continuous variables, although most analyses did not reach statistical significance.

**Table 2:** Exposure to PM_10_, PM_2.5_, and NO_2_ and risk of PD, National Institutes of Health (NIH)-AARP Diet and Health Study, 1995–2006.

Exposure	PD/No PD	OR^*a*^ (95% CI)	OR^*b*^ (95% CI)
Quintiles of PM_2.5_ (μg/m^3^)
4.4 to < 10.8	300/673	1.00 (Referent)	1.00 (Referent)
10.8 to < 12.3	319/652	1.11 (0.91, 1.34)	1.09 (0.90, 1.33)
12.3 to < 13.8	305/672	1.03 (0.85, 1.25)	1.02 (0.84, 1.23)
13.8 to < 15.4	307/667	1.04 (0.86, 1.27)	1.03 (0.85, 1.25)
15.4 to 26.9	325/649	1.14 (0.94, 1.38)	1.11 (0.92, 1.35)
*p*_Trend_^*c*^		0.31	0.43
Continuous PM_2.5_ (μg/m^3^)^*d*^	1,556/3,313	1.02 (0.95, 1.10)	1.02 (0.94, 1.10)
Quintiles of PM_10_ (μg/m^3^)
14.3 to < 22.9	315/658	1.00 (Referent)	1.00 (Referent)
22.9 to < 25.1	295/679	0.91 (0.75, 1.11)	0.91 (0.75, 1.10)
25.1 to < 27.9	305/669	0.96 (0.79, 1.16)	0.96 (0.79, 1.16)
27.9 to < 33.8	317/657	1.02 (0.84, 1.23)	1.01 (0.83, 1.22)
33.8 to 65.4	324/650	1.06 (0.87, 1.28)	1.05 (0.86, 1.27)
*p*_Trend_^*c*^		0.25	0.29
Continuous PM_10_ (μg/m^3^)^*d*^	1,556/3,313	1.03 (0.97, 1.09)	1.02 (0.97, 1.09)
Quintiles of NO_2_ (ppb)
1.0 to < 7.7	312/659	1.00 (Referent)	1.00 (Referent)
7.7 to < 10.4	303/673	0.95 (0.79, 1.15)	0.96 (0.80, 1.17)
10.4 to < 13.1	311/662	0.99 (0.82, 1.20)	0.99 (0.82, 1.20)
13.1 to < 16.6	319/656	1.03 (0.86, 1.25)	1.01 (0.83, 1.22)
16.6 to 34.2	311/663	1.00 (0.82, 1.21)	0.99 (0.81, 1.20)
*p*_T__rend_^*c*^		0.76	0.96
Continuous NO_2_ (ppb)^*d*^	1,556/3,313	1.02 (0.95, 1.11)	1.01 (0.93, 1.10)
Abbreviations: CI, confidence interval; OR, odds ratio; PD, Parkinson disease; PM_2.5_, particulate matter < 2.5 μm in aerodynamic diameter; PM_10_, particulate matter < 10 μm in aerodynamic diameter. ^***a***^All adjusted for age at baseline, sex, and race. ^***b***^Additionally adjusted for education, caffeine intake, smoking status, and physical activity. ^***c***^Based on linear model through the quintile medians. ^***d***^Change per interquartile range (IQR); Exposure (IQR): PM_2.5_ (3.8 μg/m^3^), PM_10_ (8.4 μg/m^3^), NO_2_ (7.3 ppb).			

**Table 3:** Exposure to PM_10_, PM_2.5_, and NO_2_ and risk of PD, by gender and smoking status, National Institutes of Health (NIH)-AARP Diet and Health Study, 1995–2006.

By sex	Male		Female		*p*-Int
	PD/No PD	OR^*a*^ (95% CI)	PD/No PD	OR^*a*^ (95% CI)	
Quintiles of PM_2.5_ (μg/m^3^)
4.4 to < 10.8	235/500	1.00 (Referent)	65/173	1.00 (Referent)
10.8 to < 12.3	231/498	0.98 (0.78, 1.22)	88/154	1.46 (0.99, 2.18)
12.3 to < 13.8	238/485	1.04 (0.83, 1.30)	67/187	0.93 (0.62, 1.40)
13.8 to < 15.4	218/499	0.92 (0.74, 1.16)	89/168	1.38 (0.93, 2.05)
15.4 to 26.9	232/453	1.08 (0.87, 1.36)	93/196	1.20 (0.81, 1.77)	0.69
*p*_Trend_^*b*^		0.61		0.53
Continuous PM_2.5_ (μg/m^3^)^*c*^	1,154/2,435	1.01 (0.92, 1.10)	402/878	1.02 (0.88, 1.18)	0.72
Quintiles of PM_10_ (μg/m^3^)
14.3 to < 22.9	258/497	1.00 (Referent)	57/161	1.00 (Referent)
22.9 to < 25.1	222/516	0.83 (0.67, 1.04)	73/163	1.27 (0.84, 1.93)
25.1 to < 27.9	222/490	0.89 (0.72, 1.12)	83/179	1.30 (0.86, 1.95)
27.9 to < 33.8	231/462	0.98 (0.78, 1.22)	86/195	1.25 (0.83, 1.87)
33.8 to 65.4	221/470	0.92 (0.73, 1.14)	103/180	1.65 (1.11, 2.45)	0.06
*p*_Trend_^*b*^		0.95		0.02
Continuous PM_10_ (μg/m^3^)^*c*^	1,154/2,435	1.00 (0.94, 1.07)	402/878	1.09 (0.98, 1.23)	0.22
Quintiles of NO_2_ (ppb)
1.0 to < 7.7	243/498	1.00 (Referent)	69/161	1.00 (Referent)
7.7 to < 10.4	238/504	0.98 (0.79, 1.22)	65/169	0.89 (0.59, 1.35)
10.4 to < 13.1	222/495	0.92 (0.73, 1.14)	89/167	1.29 (0.88, 1.92)
13.1 to < 16.6	239/479	1.01 (0.81, 1.26)	80/177	1.04 (0.70, 1.55)
16.6 to 34.2	212/459	0.94 (0.75, 1.18)	99/204	1.13 (0.77, 1.66)	0.39
*p*_Trend_^*b*^		0.71		0.42
Continuous NO_2_ (ppb)^*c*^	1,154/2,435	1.00 (0.91, 1.10)	402/878	1.06 (0.90, 1.24)	0.61

By smoking status	Never smokers		Ever smokers		*p*-Int
	PD/No PD	OR^*a*^ (95% CI)	PD/No PD	OR^*a*^ (95% CI)	
Quintiles of PM_2.5_ (μg/m^3^)
4.4 to < 10.8	106/212	1.00 (Referent)	193/453	1.00 (Referent)
10.8 to < 12.3	120/236	1.03 (0.75, 1.43)	194/411	1.12 (0.88, 1.43)
12.3 to < 13.8	136/243	1.16 (0.85, 1.60)	166/421	0.94 (0.73, 1.20)
13.8 to < 15.4	156/231	1.38 (1.01, 1.89)	149/427	0.83 (0.64, 1.07)
15.4 to 26.9	154/243	1.29 (0.94, 1.76)	165/398	0.99 (0.77, 1.27)	0.13
*p*_Trend_^*b*^		0.04		0.36
Continuous PM_2.5_ (μg/m^3^)^*c*^	672/1,165	1.09 (0.97, 1.22)	867/2,110	0.95 (0.86, 1.06)	0.27
Quintiles of PM_10_ (μg/m^3^)
14.3 to < 22.9	113/213	1.00 (Referent)	198/437	1.00 (Referent)
22.9 to < 25.1	129/257	0.97 (0.71, 1.33)	164/417	0.89 (0.69, 1.14)
25.1 to < 27.9	145/211	1.32 (0.96, 1.81)	156/449	0.79 (0.61, 1.01)
27.9 to < 33.8	142/241	1.14 (0.83, 1.56)	171/409	0.94 (0.74, 1.21)
33.8 to 65.4	143/243	1.13 (0.83, 1.55)	178/398	1.01 (0.79, 1.30)	0.97
*p*_Trend_^*b*^		0.51		0.41
Continuous PM_10_ (μg/m^3^)^*c*^	672/1,165	1.03 (0.94, 1.13)	867/2,110	1.02 (0.94, 1.10)	0.91
Quintiles of NO_2_ (ppb)
1.0 to < 7.7	121/226	1.00 (Referent)	188/422	1.00 (Referent)
7.7 to < 10.4	127/207	1.17 (0.85, 1.60)	171/461	0.85 (0.66, 1.09)
10.4 to < 13.1	138/228	1.14 (0.84, 1.55)	169/428	0.89 (0.70, 1.15)
13.1 to < 16.6	140/268	1.01 (0.74, 1.37)	178/380	1.06 (0.83, 1.36)
16.6 to 34.2	146/236	1.18 (0.87, 1.61)	161/419	0.87 (0.67, 1.12)	0.69
*p*_Trend_^*b*^		0.56		0.71
Continuous NO_2_ (ppb)^*c*^	672/1,165	1.02 (0.90, 1.15)	867/2,110	1.01 (0.91, 1.13)	0.86
Abbreviations: CI, confidence interval; OR, odds ratio; PD, Parkinson disease; *p*-Int, *p*-Interaction between pollutant exposures and sex (top portion of table) and smoking (bottom portion of table); PM_2.5_, particulate matter < 2.5 μm in aerodynamic diameter; PM_10_, particulate matter < 10 μm in aerodynamic diameter. ^***a***^Adjusted for age at baseline, sex (except in sex-stratified analyses), race, education, caffeine intake, smoking status (except in smoking-stratified analyses), and physical activity. ^***b***^Based on linear model through the quintile medians. ^***c***^Change per interquartile range.					

**Table 4:** Exposure to PM_10_, PM_2.5_, and NO_2_ and risk of PD among female non-smokers (*N* = 617), National Institutes of Health (NIH)-AARP Diet and Health Study, 1995–2006.

Exposure	PD/No PD	OR^*a*^ (95% CI)	OR^*b*^ (95% CI)	*p*-Int
Quintiles of PM_2.5_ (μg/m^3^)
4.4 to < 10.8	27/69	1.00 (Referent)	1.00 (Referent)
10.8 to < 12.3	44/72	1.76 (0.96, 3.23)	2.01 (1.09, 3.72)
12.3 to < 13.8	37/84	1.34 (0.72, 2.49)	1.65 (0.86, 3.19)
13.8 to < 15.4	58/73	2.38 (1.32, 4.31)	3.19 (1.68, 6.08)
15.4 to 26.9	59/94	1.79 (1.01, 3.17)	1.90 (1.05, 3.44)	0.16
*p*_Trend_^*c*^		0.05	0.05
Continuous PM_2.5_ (μg/m^3^)^*d*^	225/392	1.14 (0.95, 1.38)	1.12 (0.93, 1.36)	0.09
Quintiles of PM_10_ (μg/m^3^)
14.3 to < 22.9	24/63	1.00 (Referent)	1.00 (Referent)
22.9 to < 25.1	39/81	1.43 (0.77, 2.68)	1.44 (0.77, 2.70)
25.1 to < 27.9	50/78	1.81 (0.99, 3.32)	1.78 (0.97, 3.29)
27.9 to < 33.8	51/90	1.65 (0.90, 3.00)	1.51 (0.80, 2.88)
33.8 to 65.4	61/80	2.34 (1.29, 4.26)	1.99 (0.93, 4.26)	0.47
*p*_Trend_^*c*^		0.01	0.16
Continuous PM_10_ (μg/m^3^)^*d*^	225/392	1.18 (1.01, 1.39)	1.09 (0.88, 1.36)	0.13
Quintiles of NO_2_ (ppb)
1.0 to < 7.7	42/76	1.00 (Referent)	1.00 (Referent)
7.7 to < 10.4	31/72	0.81 (0.45, 1.46)	0.83 (0.46, 1.51)
10.4 to < 13.1	46/60	1.47 (0.84, 2.57)	1.49 (0.83, 2.68)
13.1 to < 16.6	46/93	1.00 (0.58, 1.70)	0.91 (0.49, 1.69)
16.6 to 34.2	60/91	1.35 (0.80, 2.28)	1.18 (0.63, 2.21)	0.63
*p*_Trend_^*c*^		0.19	0.54
Continuous NO_2_ (ppb)^*d*^	225/392	1.11 (0.89, 1.38)	1.00 (0.77, 1.31)	0.71
Abbreviations: CI, confidence interval; OR, odds ratio; *p*-Int, *p*-Interaction between pollutant exposures and region; PD, Parkinson disease. ^***a***^Adjusted for age at baseline, race, education, caffeine intake, and physical activity. ^***b***^Additionally adjusted for region (northeast, midwest, west, and south). ^***c***^Based on linear model through the quintile medians. ^***d***^Change per interquartile range.				

We performed additional sensitivity analyses stratified by moving status among female nonsmokers (see Table S3). Women who maintained the same residential latitude and longitude coordinates between baseline and follow-up surveys were classified as nonmovers, and those with a change in either latitude or longitude were classified as movers. Among nonmovers, the risk for PD increased monotonically with increasing exposure to PM_10_ concentrations (OR_Q5 vs. Q1_ = 2.67; 95% CI: 1.20, 5.96; *p*-trend = 0.01). However, no statistically significant trends were found among movers.

Similar results were observed in the sensitivity analyses restricted to physician-confirmed cases. Increased exposure to PM_10_ was associated with a higher risk of PD among women (OR_Q5 vs. Q1_ = 2.12; 95% CI: 1.19, 3.80; *p*-trend = 0.02), and increased exposure to PM_2.5_ was associated with greater PD risk among never smokers (OR_Q5 vs. Q1_ = 1.26; 95% CI: 0.83, 1.92; *p*-trend = 0.19). In the analyses of female never smokers, elevated PD risk was associated with the top two quintiles of PM_2.5_ (OR_Q4 vs. Q1_ = 2.69; 95% CI: 1.24, 5.85 and OR_Q5 vs. Q1_ = 1.63; 95% CI: 0.76, 3.54; *p*-trend = 0.31) and with the top quintile of PM_10_ (OR_Q5 vs. Q1_ = 2.17; 95% CI: 0.97, 4.82; *p*-trend = 0.12). No other statistically significant associations were observed.

## Discussion

In this large, nested case–control study, we did not find strong evidence for an association between exposures to ambient PM_10_, PM_2.5_, or NO_2_ concentrations and risk for PD in older adults. However, subgroup analyses suggested that female nonsmokers exposed to higher concentrations of PM_10_ or PM_2.5_ may have an increased risk for PD.

The deleterious effects of ambient air pollution on cardiovascular and pulmonary outcomes have been well documented (Block et al. 2012; Brook et al. 2004), and recent evidence has provided key insights into the impact of these adverse effects on the brain (Block et al. 2012). As a complex mixture, air pollution likely exerts adverse effects on the brain through multiple interrelated mechanisms that may subsequently lead to neurodegenerative diseases such as PD (Block and Calderón-Garcidueñas 2009). Systemic inflammation triggered by pollutant-inflamed peripheral organs including the lung, gut, and cardiovascular system may contribute to the disruption of olfactory, respiratory, and blood–brain barriers (Block et al. 2012). These cascading events may work synergistically to enhance the access of pollutants and systemic inflammatory mediators to the central nervous system (CNS), leading to neuroinflammation and neurotoxicity (Block and Calderón-Garcidueñas 2009; Block et al. 2012; Calderón-Garcidueñas et al. 2013). Chronic exposures to varying sizes and compositions of PM have been shown to induce pathological hallmarks of PD, including neuroinflammation, aggregation of α-synuclein, and neuronal oxidative stress (Block et al. 2012; Calderón-Garcidueñas et al. 2013), even in early childhood (Calderón-Garcidueñas et al. 2013). Experimental data have also shown PD neuropathology in animals exposed to concentrated urban PM or diesel exhaust, including significant reduction of dopaminergic neurons in the substantia nigra (Veronesi et al. 2005), elevated α-synuclein in the midbrain (Levesque et al. 2011), and activation of unfolded protein response in the striatum (Guerra et al. 2013). As a route of entry for air pollutants to the brain, evidence from animal studies suggests that the nasal cavity likely provides a direct transport pathway through which inhaled PM gains entry into the olfactory bulb and subsequently into the brain and brainstem (Block et al. 2012; Calderón-Garcidueñas et al. 2010, 2015). Interestingly, lesions of the olfactory bulb have been postulated as one of the earliest pathologic features of PD (Braak et al. 2003), with supporting epidemiologic evidence showing olfactory deficit as one of the most important prodromal symptoms of PD (Langston 2011). Alternatively, particles being cleared from the deep lung via the mucociliary escalator or those too large to enter the lung are swallowed and eventually end up in the gut. Thus, the sensory afferent of the dorsal vagus nerve located in the gastrointestinal tract has been postulated as another potential route of entry for air pollutants because of its ability to communicate directly with brain stem neurons (Block et al. 2012). Both the enteric plexus and the olfactory bulb have been proposed as key routes through which a neurotropic pathogen initiates the pathological process underlying sporadic PD (Hawkes et al. 2007). Indeed, Lewy body pathology has been found to start early in the enteric plexus of the stomach and in the olfactory bulb (Hawkes et al. 2007). Therefore, investigations of air pollution and PD may not only improve our understanding of the pollutant effects on PD risk but also elucidate underlying mechanisms involved in PD development and progression.

However, few epidemiologic studies have evaluated exposures to ambient air pollution in relation to PD risk, and the results have been mixed. In their study of U.S. Medicare Part A beneficiaries, Willis et al. (2010) found significantly increased incidence of PD among participants living in urban counties with high cumulative industrial release of manganese (> 75th percentile), as reported in the U.S. EPA Toxic Release Inventory database. A Canadian case–control study of two urban cities using PD cases from administrative data sets also reported a significant association of PD with exposures to ambient levels of manganese defined as manganese fraction in total suspended particulate (Finkelstein and Jerrett 2007). However, this study did not find any associations between exposures to urban traffic and neighborhood levels of NO_2_, markers of traffic-generated air pollution, and the risk for PD (Finkelstein and Jerrett 2007). The Nurses’ Health Study recently reported a positive association between airborne mercury exposure and PD, particularly among never smokers and among participants living in urban counties (Palacios et al. 2014b). However, evaluation of PM exposures in the same prospective cohort found no evidence to support an effect of air pollution on PD risk (Palacios et al. 2014a). A more recent study among farmers in Iowa and North Carolina reported borderline positive associations of PD with exposures to ozone and PM_2.5_ in North Carolina, but not in Iowa (Kirrane et al. 2015). A recent publication by Ritz et al. (2016) reported an increased risk of PD with long-term exposure to NO_2_ in a Danish population, specifically among those born or living in the capital city or in provincial towns. Only two of these six existing epidemiologic studies investigated potential effect modification by sex, and in both studies, no significant sex differences were observed (Finkelstein and Jerrett 2007; Ritz et al. 2016).

We planned *a priori* analyses stratified by sex and by smoking status because sex differences exist in both PD prevalence and risk profiles (Haaxma et al. 2007) and because smoking is an important risk factor for PD (Chen et al. 2010). Moreover, a number of epidemiologic studies of cardiovascular and respiratory health outcomes showed stronger adverse effects of air pollutants among women, particularly in studies of older adults and those using residential exposure assessment (Clougherty 2010). Interestingly, in our analysis, a higher risk of PD was observed among women and never smokers exposed to greater levels of PM. Although we cannot completely exclude the possibility that this finding is by chance, the observed modification is likely attributable, at least in part, to differences in exposure patterns and biological responses to air pollution between men and women (Clougherty 2010). Indeed, physiological differences between the sexes, such as hormonal status and body size, have been shown to influence the biological transport of environmental toxicants (Clougherty 2010). Other biological traits such as lung size (Kim and Hu 1998, 2006), deposition of inhaled particles (Kim and Hu 2006), blood-gas barrier permeability (Bräuner et al. 2009), and inflammation (Hoffmann et al. 2009) also differ by sex. Additionally, sex-related differences in occupation and in lifestyle factors such as smoking and alcohol consumption also likely play a role in differential exposure patterns between men and women (Clougherty 2010; Oiamo and Luginaah 2013). The evidence of sex differences in susceptibility to air pollution remains unknown, and further investigation and reporting of sex-stratified results will be informative and may provide some insights into possible biological mechanisms.

The precise reasons for the lack of clear associations of PD with PM_2.5_ among women and with PM_10_ among never smokers are unknown but may reflect random or exposure assessment errors, or they may indicate residual confounding rather than a lack of neurotoxic effects. Interestingly, in analyses restricted to female never smokers, both PM_2.5_ and PM_10_ were significantly associated with higher risk of PD. Indeed, both PM_2.5_ and PM_10_ have been shown to exert neurotoxic effects on the brain. In addition to its well-documented effects on neuroinflammation and oxidative stress (Block and Calderón-Garcidueñas 2009), PM_2.5_ exposure may disrupt the blood–brain barrier (Liu et al. 2015). Although much research has focused on small particles, recent data suggest that larger particles may also have neurotoxic effects. For instance, one *in vivo* study suggested that the peripheral inflammatory response to PM_10_ exposure in mice may trigger adverse effects in the brain (Farina et al. 2013). Another murine study reported evidence of neuroinflammation, oxidative stress and unfolded protein responses in striatum activated by inhalation exposure to coarse PM (Guerra et al. 2013).

The present study has a number of notable strengths. A key strength was the use of finely resolved, validated, national air pollutant models based on residential address. Our study population was geographically diverse, which allowed the assessment of a wide range of exposure gradients. Our relatively large sample size included > 1,000 male and female PD cases. Furthermore, we conducted a number of sensitivity analyses; for example, we limited analyses to physician-confirmed cases, and we stratified analyses by region of residency. The results from these sensitivity analyses were consistent with the primary findings.

Our study also has several limitations. First, measurement of long-term air pollution exposure is prone to misclassification. In the present study, we used the outdoor concentration at the baseline residential address to characterize exposure. We do not have precise information on the concentrations in the microenvironments in which cases and controls spent their time. The specific years used for predicting the different pollutants were based on the availability of the modeled air pollutant data. For this reason, the years of exposure assessment for PM_2.5_ and NO_2_ were after disease diagnosis. However, we did not expect that the particular prediction year being modeled would have made a substantial difference; for both PM_2.5_ and NO_2_, the correlations between predictions in adjacent years are high (*r* > 0.96). Nevertheless, because of declining PM and NO_2_ levels since the 1990s, it may be possible that the “baseline” exposures to PM_2.5_ and NO_2_ were systematically underestimated. Second, the pollutant estimates that we have are only representative of a portion of the participants’ exposure in adulthood, but earlier time frames may be at least as important. The time frame, severity, and type of air pollution exposures during an individual’s lifetime may be critical for evaluating deleterious effects of air pollution on PD pathology. Alarmingly, children residing in the severely polluted Mexico City Metropolitan Area (MCMA) already exhibit symptoms seen in the premotor stage of PD, including olfactory disturbances and severe autonomic dysfunction (Calderón-Garcidueñas et al. 2013). The presence of α-synuclein associated with PD pathology was also detected in the olfactory bulb, midbrain, and lower brainstem of MCMA children (Calderón-Garcidueñas et al. 2013). Given the long duration of the preclinical stage of PD before overt motor symptoms appear, cumulative lifetime exposure assessments spanning from early childhood to adulthood would be ideal. Third, we only collected residential addresses, and data on potential exposures to pollutants in the workplace were not available. Another limitation is that PD diagnosis was only asked once at the follow-up survey with categorical choices for the year of diagnosis; therefore, some cases were inevitably missed during the follow-up, and we were unable to perform more desirable risk-set sampling for controls. Further, PD case identification was based on self-reports. It is inevitable that some cases were missed and that some were misdiagnosed. However, our validation study confirmed 88% of self-reported diagnoses among those with medical information available, and we excluded cases with erroneous reports from the analysis. Finally, although our cohort was relatively large, we still had only modest numbers of PD cases in some analyses; together with potential measurement errors in exposure and outcome assessments, this small number of cases may have limited our ability to detect moderate associations.

## Conclusion

In conclusion, although there was no statistically significant association between ambient air pollution and PD risk in this cohort of older adults overall, we found a higher risk of PD among both women and never smokers with exposures to high levels of PM_2.5_ and PM_10._ These findings warrant further investigation.

## Supplemental Material

(341 KB) PDF
