# Supplementary material for: Ambient Air Pollution Exposures and Risk of Parkinson Disease
Source: Environ Health Perspect. 2016 Jun 10;124(11):1759–65. doi: 10.1289/EHP135 (PMC5089873; doi:10.1289/EHP135)
Supplement: (341 KB) PDF [file EHP135.s001.acco.pdf]

Environ Health Perspect
DOI: 10.1289/EHP135

Note to readers with disabilities: *EHP* strives to ensure that all journal content is accessible to all readers. However, some figures and Supplemental Material published in *EHP* articles may not conform to 508 standards due to the complexity of the information being presented. If you need assistance accessing journal content, please contact ehp508@niehs.nih.gov. Our staff will work with you to assess and meet your accessibility needs within 3 working days.

Supplemental Material

Ambient Air Pollution Exposures and Risk of Parkinson Disease

Rui Liu, Michael T. Young, Jiu-Chiuan Chen, Joel D. Kaufman, and Honglei Chen

Table of Contents

Table S1. Exposure to PM_{10}, $PM_{2.5}$, and NO_2 and risk of PD, by gender and smoking status, and by US regions, NIH-AARP Diet and Health Study, 1995-2006
Table S2. Exposure to PM_{10}, $PM_{2.5}$, and NO_2 and risk of PD among female non-smokers (N=617), by US regions, NIH-AARP Diet and Health Study, 1995-2006
Table S3. Exposure to PM_{10}, $PM_{2.5}$, and NO_2 and risk of PD among female non-smokers (N=617), by moving status during baseline and study follow-up

Table S1. Exposure to PM_{10}, $PM_{2.5}$, and NO_2 and risk of PD, by gender and smoking status, and by US regions, NIH-AARP Diet and Health Study, 1995-2006

	Northeast			West			South			P-int[d]
**By sex:**	**PD / No PD**	**OR[a]**	**95% CI**	**PD / No PD**	**OR[a]**	**95% CI**	**PD / No PD**	**OR[a]**	**95% CI**	
**Male**										
Tertiles of $PM_{2.5}$ (μg/m^3)										
4.4- <11.9	15/36	1.00	Referent	164/329	1.00	Referent	208/452	1.00	Referent	
11.9- <14.3	210/397	1.34	0.70, 2.55	74/176	0.85	0.60, 1.18	95/237	0.90	0.67, 1.22	
14.3- 26.9	115/246	1.18	0.61, 2.30	119/248	0.96	0.72, 1.29	93/183	1.09	0.80, 1.48	0.68
P_{trend}[b]		0.77				0.73		0.75		
Continuous $PM_{2.5}$ (μg/m^3)[c]	340/679	1.00	0.68, 1.48	357/753	0.98	0.88, 1.10	396/872	1.04	0.87, 1.25	0.87
Tertiles of PM_{10} (μg/m^3)										
14.3- <24.4	148/290	1.00	Referent	21/50	1.00	Referent	218/463	1.00	Referent	
24.4- <29.4	147/322	0.93	0.69, 1.25	63/124	1.29	0.70, 2.37	139/323	0.90	0.70, 1.17	
29.4- 65.4	45/67	1.37	0.87, 2.14	273/579	1.21	0.70, 2.08	39/86	0.93	0.61, 1.42	0.74
P_{trend}[b]		0.21				0.98		0.63		
Continuous PM_{10} (μg/m^3)[c]	340/679	1.18	0.83, 1.67	357/753	1.01	0.91, 1.12	396/872	0.87	0.65, 1.15	0.61
Tertiles of NO_2 (ppb)										
1.0- <9.5	48/87	1.00	Referent	78/147	1.00	Referent	267/580	1.00	Referent	
9.5- <14.2	152/303	0.88	0.58, 1.33	100/226	0.85	0.59, 1.23	122/268	0.97	0.75, 1.26	
14.2- 34.2	140/289	0.84	0.55, 1.28	179/380	0.87	0.62, 1.22	7/24	0.64	0.26, 1.51	0.93
P_{trend}[b]		0.47				0.53			0.47	
Continuous NO_2 (ppb)[c]	340/679	1.00	0.80, 1.26	357/753	0.99	0.86, 1.15	396/872	0.92	0.68, 1.24	0.77
**Female**										
Tertiles of $PM_{2.5}$ (μg/m^3)										
4.4- <11.9	7/12	1.00	Referent	64/116	1.00	Referent	49/152	1.00	Referent	
11.9- <14.3	52/136	0.71	0.25, 2.02	40/74	0.97	0.58, 1.61	32/79	1.19	0.68, 2.08	
14.3- 26.9	42/91	0.96	0.33, 2.83	54/99	0.97	0.61, 1.57	40/73	1.92	1.12, 3.31	0.40
P_{trend}[b]		0.52			0.91			0.02		
Continuous $PM_{2.5}$ (μg/m^3)[c]	101/239	1.27	0.59, 2.71	158/289	0.97	0.82, 1.16	121/304	1.32	0.93, 1.86	0.45

Tertiles of PM_{10} ($\mu g/m^3$)										
14.3- <24.4	35/85	1.00	Referent	13/15	1.00	Referent	46/160	1.00	Referent	
24.4- <29.4	47/112	1.02	0.58, 1.79	22/50	0.45	0.18, 1.14	59/115	1.94	1.19, 3.15	
29.4- 65.4	19/42	1.26	0.58, 2.74	123/224	0.58	0.26, 1.31	16/29	2.23	1.04, 4.79	0.06
P_{trend}[b]		0.55				0.81		0.02		
Continuous PM_{10} ($\mu g/m^3$)[c]	101/239	1.25	0.63, 2.50	158/289	0.99	0.83, 1.18	121/304	1.48	0.86, 2.56	0.58
Tertiles of NO_2 (ppb)										
1.0- <9.5	7/21	1.00	Referent	25/56	1.00	Referent	76/195	1.00	Referent	
9.5- <14.2	46/86	1.80	0.68, 4.81	50/77	1.44	0.77, 2.69	40/100	1.05	0.65, 1.71	
14.2- 34.2	48/132	1.22	0.46, 3.25	83/156	1.13	0.64, 2.00	5/9	1.84	0.53, 3.67	0.60
P_{trend}[b]		0.53				0.96			0.48	
Continuous NO_2 (ppb)[c]	101/239	0.97	0.63, 1.49	158/289	0.95	0.75, 1.21	121/304	1.23	0.71, 2.15	0.58
	**Northeast**			**West**			**South**			
**By smoking status:**	**PD / No PD**	**OR[a]**	**95% CI**	**PD / No PD**	**OR[a]**	**95% CI**	**PD / No PD**	**OR[a]**	**95% CI**	
**Never Smokers**										
Tertiles of $PM_{2.5}$ ($\mu g/m^3$)										
4.4- <11.9	6/19	1.00	Referent	88/164	1.00	Referent	90/186	1.00	Referent	
11.9- <14.3	115/204	1.86	0.70, 4.93	59/95	1.13	0.74, 1.74	48/99	1.06	0.69, 1.65	
14.3- 26.9	78/121	2.15	0.79, 5.89	81/131	1.09	0.74, 1.62	65/91	1.52	1.00, 2.30	0.48
P_{trend}[b]		0.16				0.63		0.05		
Continuous $PM_{2.5}$ ($\mu g/m^3$)[c]	199/344	1.48	0.87, 2.51	228/390	1.01	0.88, 1.16	203/376	1.21	0.93, 1.56	0.31
Tertiles of PM_{10} ($\mu g/m^3$)										
14.3- <24.4	76/141	1.00	Referent	19/26	1.00	Referent	95/196	1.00	Referent	
24.4- <29.4	95/159	1.11	0.75, 1.67	38/66	0.80	0.38, 1.65	84/145	1.21	0.83, 1.77	
29.4- 65.4	28/44	1.16	0.64, 2.10	171/298	0.76	0.40, 1.44	24/35	1.45	0.80, 2.62	0.19
P_{trend}[b]		0.61				0.50		0.19		
Continuous PM_{10} ($\mu g/m^3$)[c]	199/344	1.42	0.85, 2.35	228/390	1.01	1.88, 1.17	203/376	1.21	0.78, 1.86	0.22
Tertiles of NO_2 (ppb)										
1.0- <9.5	16/37	1.00	Referent	44/64	1.00	Referent	143/246	1.00	Referent	

9.5- <14.2	98/142	1.79	0.92, 3.51	67/116	0.84	0.51, 1.40	59/121	0.82	0.55, 1.20	
14.2- 34.2	85/165	1.19	0.61, 2.35	117/210	0.74	0.47, 1.18	1/9	0.19	0.02, 1.52	0.64
P_{trend}[b]		0.40				0.21			0.09	
Continuous NO_2 (ppb)[c]	199/344	1.01	0.73, 1.39	228/390	0.95	0.78, 1.14	203/376	0.87	0.56, 1.35	0.85
**Ever Smokers**										
Tertiles of $PM_{2.5}$ ($\mu g/m^3$)										
4.4- <11.9	16/28	1.00	Referent	138/277	1.00	Referent	166/412	1.00	Referent	
11.9- <14.3	144/324	0.80	0.41, 1.53	54/151	0.74	0.50, 1.07	76/211	0.90	0.65, 1.25	
14.3- 26.9	77/214	0.66	0.34, 1.32	90/212	0.86	0.62, 1.20	66/163	1.03	0.73, 1.46	0.68
P_{trend}[b]		0.17				0.30		0.98		
Continuous $PM_{2.5}$ ($\mu g/m^3$)[c]	237/566	0.76	0.48, 1.20	282/640	0.94	0.83, 1.08	308/786	1.03	0.84, 1.28	0.65
Tertiles of PM_{10} ($\mu g/m^3$)										
14.3- <24.4	104/231	1.00	Referent	15/39	1.00	Referent	167/419	1.00	Referent	
24.4- <29.4	97/271	0.84	0.60, 1.18	47/106	1.42	0.70, 2.91	111/289	0.98	0.73, 1.31	
29.4- 65.4	36/64	1.35	0.81, 2.25	220/495	1.38	0.73, 2.63	30/78	0.92	0.58, 1.48	0.87
P_{trend}[b]			0.31			0.60		0.73		
Continuous PM_{10} ($\mu g/m^3$)[c]	237/566	0.97	0.64, 1.48	282/640	1.00	0.88, 1.13	308/786	0.85	0.61, 1.18	0.90
Tertiles of NO_2 (ppb)										
1.0- <9.5	36/70	1.00	Referent	59/136	1.00	Referent	196/518	1.00	Referent	
9.5- <14.2	99/243	0.77	0.48, 1.24	81/183	1.00	0.66, 1.51	101/245	1.08	0.81, 1.44	
14.2- 34.2	102/253	0.79	0.49, 1.28	142/321	1.00	0.69, 1.46	11/23	1.35	0.61, 2.97	0.65
P_{trend}[b]			0.32			0.32			0.43	
Continuous NO_2 (ppb)[c]	237/566	0.94	0.73, 1.20	282/640	0.99	0.83, 1.17	308/786	1.07	0.76, 1.50	0.97

Abbreviations: CI, confidence interval; OR, odds ratio; PD, Parkinson disease

[a] Adjusted for age at baseline, sex (except in sex stratified analyses), race, education, caffeine intake, smoking status (except in smoking stratified analyses), and physical activity.

[b] Based on liner model through the tertile medians.

[c] Change per interquartile range.

[d] P-interaction between pollutant exposures and region.

Table S2. Exposure to PM_{10}, $PM_{2.5}$, and NO_2 and risk of PD among female non-smokers (N=617), by US regions, NIH-AARP Diet and Health Study, 1995-2006

	Northeast			West			South		
**Exposure**	**PD / No PD**	**OR[a]**	**95% CI**	**PD / No PD**	**OR[a]**	**95% CI**	**PD / No PD**	**OR[a]**	**95% CI**
Tertiles of $PM_{2.5}$ ($\mu g/m^3$)									
4.4- <11.9	3/6	1.00	Referent	31/49	1.00	Referent	17/64	1.00	Referent
11.9- <14.3	28/62	1.43	0.27, 7.43	26/33	1.33	0.62, 2.83	21/36	2.23	0.99, 5.03
14.3- 26.9	23/40	2.23	0.40, 12.50	36/47	1.23	0.62, 2.44	26/31	3.09	1.40, 6.83
P_{trend}[b]			0.16			0.54			0.005
Continuous $PM_{2.5}$ ($\mu g/m^3$)[c]	54/108	1.72	0.59, 5.01	93/129	1.04	0.83, 1.32	64/131	1.81	1.11, 2.93
Tertiles of PM_{10} ($\mu g/m^3$)									
14.3- <24.4	18/38	1.00	Referent	5/5	1.00	Referent	21/70	1.00	Referent
24.4- <29.4	28/56	1.01	0.44, 2.33	12/20	0.65	0.14, 2.99	32/49	2.56	1.25, 5.27
29.4- 65.4	8/14	2.13	0.60, 7.53	76/104	0.75	0.19, 2.96	11/12	3.56	1.22, 10.36
P_{trend}[b]			0.18			0.99		0.01	
Continuous PM_{10} ($\mu g/m^3$)[c]	54/108	2.16	0.70, 6.72	93/129	1.06	0.83, 1.36	64/131	1.98	0.90, 4.34
Tertiles of NO_2 (ppb)									
1.0- <9.5	2/9	1.00	Referent	13/22	1.00	Referent	46/88	1.00	Referent
9.5- <14.2	26/40	4.85	0.83, 28.52	28/30	2.14	0.82, 5.57	17/40	0.74	0.36, 1.52
14.2- 34.2	26/59	3.21	0.55, 18.87	52/77	1.34	0.57, 3.15	1/3	1.19	1.10, 14.45
P_{trend}[b]			0.85			0.96			0.53
Continuous NO_2 (ppb)[c]	54/108	1.49	0.70, 3.18	93/129	0.96	0.67, 1.35	64/131	0.90	0.40, 2.00

Abbreviations: CI, confidence interval; OR, odds ratio; PD, Parkinson disease

[a] Adjusted for age at baseline, race, education, caffeine intake, and physical activity.

[b] Based on liner model through the tertile medians.

[c] Change per interquartile range.

Table S3. Exposure to PM_{10}, $PM_{2.5}$, and NO_2 and risk of PD among female non-smokers (N=617), by moving status during baseline and study follow-up

	Non-movers			Movers		
	PD / No PD	OR[a]	95% CI	PD / No PD	OR[a]	95% CI
Quintiles of $PM_{2.5}$ (μg/m^3)						
4.4- <10.8	17/45	1.00	Referent	10/24	1.00	Referent
10.8- <12.3	22/43	1.58	0.71, 3.50	22/29	2.04	0.76, 5.46
12.3- <13.8	21/59	1.17	0.52, 2.61	16/25	2.12	0.73, 6.19
13.8- <15.4	32/49	2.09	0.97, 4.48	26/24	3.30	1.20, 9.09
15.4- 26.9	35/61	1.86	0.89, 3.91	24/33	1.93	0.73, 5.12
P_{trend}[b]		0.07			0.23	
Continuous $PM_{2.5}$ (μg/m^3)[c]	127/257	1.14	0.89, 1.45	98/135	1.21	0.87, 1.68
Quintiles of PM_{10} (μg/m^3)						
14.3- <22.9	14/43	1.00	Referent	10/20	1.00	Referent
22.9- <25.1	18/50	1.31	0.56, 3.04	21/31	1.34	0.48, 3.73
25.1- <27.9	28/54	1.80	0.82, 3.94	22/24	1.95	0.70, 5.46
27.9- <33.8	35/60	2.07	0.95, 4.47	16/30	0.99	0.35, 2.88
33.8- 65.4	32/50	2.67	1.20, 5.96	29/30	2.12	0.79, 5.68
P_{trend}[b]		0.01			0.20	
Continuous PM_{10} (μg/m^3)[c]	127/257	1.20	0.98, 1.47	98/135	1.20	0.90, 1.59
Quintiles of NO_2 (ppb)						
1.0- <7.7	30/49	1.00	Referent	12/27	1.00	Referent
7.7- <10.4	15/43	0.63	0.29, 1.38	16/29	1.34	0.51, 3.55
10.4- <13.1	24/43	0.99	0.49, 2.00	22/17	3.33	1.19, 9.35
13.1- <16.6	22/59	0.69	0.34, 1.39	24/34	1.85	0.74, 4.65
16.6- 34.2	36/63	1.09	0.57, 2.11	24/28	2.05	0.80, 5.25
P_{trend}[b]		0.62			0.16	
Continuous NO_2 (ppb)[c]	127/257	1.03	0.76, 1.41	98/135	1.24	0.87, 1.75

Abbreviations: CI, confidence interval; OR, odds ratio; PD, Parkinson disease
[a] Adjusted for age at baseline, race, education, caffeine intake, and physical activity.
[b] Based on liner model through the quintile medians.
[c] Change per interquartile range.
